# No Evidence for Immune Priming in Ants Exposed to a Fungal Pathogen

**DOI:** 10.1371/journal.pone.0035372

**Published:** 2012-04-16

**Authors:** Anabelle Reber, Michel Chapuisat

**Affiliations:** Department of Ecology and Evolution, University of Lausanne, Lausanne, Switzerland; Aarhus University, Denmark

## Abstract

There is accumulating evidence that invertebrates can acquire long-term protection against pathogens through immune priming. However, the range of pathogens eliciting immune priming and the specificity of the response remain unclear. Here, we tested if the exposure to a natural fungal pathogen elicited immune priming in ants. We found no evidence for immune priming in *Formica selysi* workers exposed to *Beauveria bassiana*. The initial exposure of ants to the fungus did not alter their resistance in a subsequent challenge with the same fungus. There was no sign of priming when using homologous and heterologous combinations of fungal strains for exposure and subsequent challenges at two time intervals. Hence, within the range of conditions tested, the immune response of this social insect to the fungal pathogen appears to lack memory and strain-specificity. These results show that immune priming is not ubiquitous across pathogens, hosts and conditions, possibly because of immune evasion by the pathogen or efficient social defences by the host.

## Introduction

The immune system of invertebrates has long been assumed to lack memory and specificity. This view has changed, as recent studies have documented that a primary exposure of invertebrates to pathogens increased their resistance to a later pathogenic challenge, a phenomenon called “immune priming" [1]–[3]. Immune priming in invertebrates may confer long-term protection against specific pathogens, thus being functionally similar to the acquired immunity of vertebrates [3]–[5]. However, the generality, adaptive significance and mechanistic basis of invertebrate immune priming remain unclear [6].

Data on immune priming are still scarce, so that it is difficult to assess if the occurrence of priming is universal or restricted to specific combinations of hosts, pathogens and experimental conditions [3]. Immune priming has been documented in various insect species exposed to bacteria [7]–[10], protozoa [11] and virus [12]. In contrast, many early studies of invertebrate immune response failed to detect memory [6], and priming was not detected in field-collected damselflies exposed to bacteria [13]. So far, evidence for individual immune priming in response to fungal pathogens are limited, with one case in termites exposed to *Metarhizium anisopliae* [7] and another one in fruit flies challenged with *Beauveria bassiana* [9].

The degree of specificity of immune priming seems variable [3]. In some experiments, immune priming was nonspecific and protected against multiple pathogens. For example, a physical stress was sufficient to increase the defences of a moth against a yeast infection [14], flour beetles inoculated with lipopolysaccharides from bacterial cell walls became more resistant to a fungal pathogen [15] and bumblebees injected with glucans from fungal cell walls showed elevated response against bacteria [16]. In other experiments, immune priming was species-specific or even strain-specific, as the protection was more efficient when the challenge involved the same pathogen species or strain as the primary exposure [8]–[10].

The duration of the protection conferred by priming may also vary. The effect of priming has typically been tested over short time periods, between three and 22 days after the first exposure [1], [8]–[12], [15]. However, the immune protection due to priming can persist after complete metamorphosis [17] and can even be transferred to the next generation, with offspring being less susceptible to pathogens that their mother or father had previously encountered [12], [18]–[23].

Overall, more empirical studies are needed to draw an accurate picture of the occurrence, duration and specificity of immune priming across invertebrates and their pathogens [3], [6]. Some classes of pathogens, such as the entomopathogenic fungi, have been little studied. Pathogens vary in their infection pathways, in the components of the immune system they trigger, as well as in their ability to evade the immune response of their hosts [2], [3], [24], [25]. Moreover, the ecology, behaviour and life-history of the hosts may also affect their immune response and sensitivity to pathogens [24], [26].

Sociality brings a novel dimension to the study of immune defences. The close co-existence of related individuals in long-lasting social groups may favour the spread of pathogens [27], [28]. However, some of the defences can be externalized and shared, thus conferring social immunity in addition to individual immunity [29]–[32]. Interestingly, two studies found that termites and ants had higher resistance to a fungal pathogen when they had been in contact with nestmates previously exposed the same pathogen [33], [34]. Moreover, ants injected with bacteria or lipopolysaccharides had a higher rate of trophallaxis and regurgitated droplet that had a higher level of antibacterial activity [35]. These results suggest that various modes of exposure to fungal or bacterial pathogens trigger an immune response that can be transmitted to nestmates. However, it remains unclear whether the exposed individuals themselves also become more resistant after the first exposure to the pathogen (individual immune priming), or if the reaction is only beneficial to nestmates (social immune priming).

Here, we used the ant *Formica selysi* and its natural fungal pathogen *B. bassiana* to test for individual or social immune priming. Specifically, we examined whether a primary exposure of groups of workers to a low dose of the fungal pathogen increased the individual resistance of these workers in a subsequent challenge with a lethal dose of the same pathogen. Because the persistence and specificity of invertebrate immune priming remain poorly understood, we challenged the ants either eight or 16 days after the beginning of the primary exposure, using homologous and heterologous combinations of two genetic strains of *B. bassiana* isolated from our study population.

## Results

**Figure 1:**
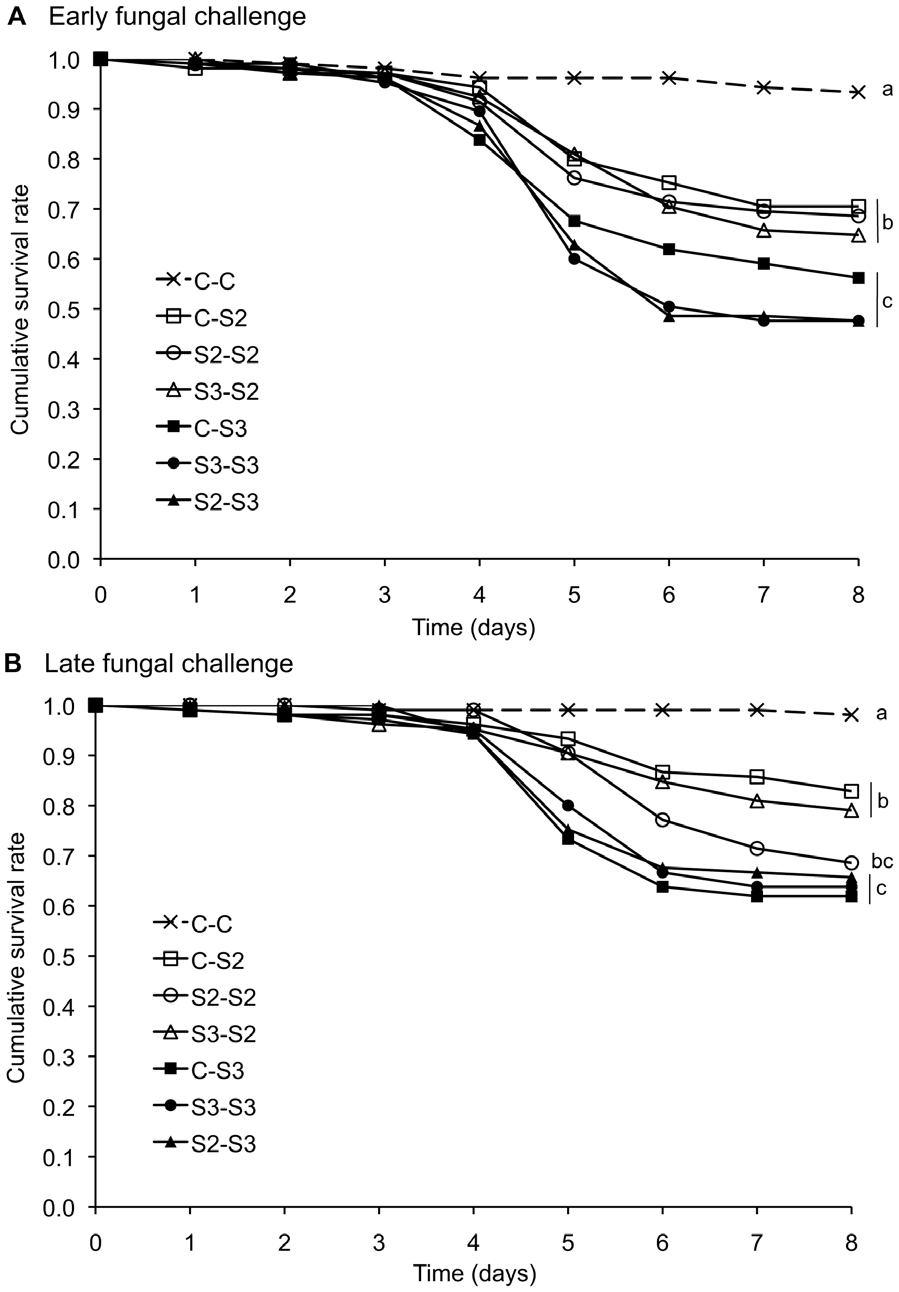
Test of immune priming in the ant *F. selysi* exposed to the fungal entomopathogen *B. bassiana*. Individual ants were challenged with a high dose of *B. bassiana* strain S2 (open symbols) or strain S3 (closed symbols) after having been initially exposed to control buffer (no priming, squares), low dose of the same strain of *B. bassiana* (homologous priming, circles), or low dose of the other strain (heterologous priming, triangles). In additional controls, the ants were exposed and “mock-challenged" with control buffer only (crosses and dashed lines). The ants were challenged either eight days (panel A, early fungal challenge) or 16 days (panel B, late fungal challenge) after the beginning of the six-day long period of primary exposure. Different letters indicate treatments that differed significantly from one another.

We found no evidence for immune priming in the ant *F. selysi* exposed to the fungal pathogen *B. bassiana*. Indeed, the initial exposure of ants to a sublethal dose of fungal spores had no significant effect on their survival when they were later challenged with a lethal dose of spores (Fig. 1 and Table 1; Effect of priming, early challenge: χ^2^ = 2.8, d.f. = 2.1, *P* = 0.3, late challenge: χ^2^ = 0.9, d.f. = 12.1, *P* = 0.7). There was no sign of priming when we used the same fungal strain for priming and challenge (Fig. 1 and Table 1; C-S2 vs S2-S2 and C-S3 vs S3-S3), nor when we used heterologous combinations of strains for priming and challenge (Fig. 1 and Table 1; C-S2 vs S3-S2 and C-S3 vs S2-S3). Moreover, there was no significant difference in survival when the ants were primed and challenged with heterologous combinations, as compared to homologous combinations (Table 1; S2-S2 vs S3-S2 and S3-S3 vs S2-S3) and no interaction between the factors “priming" and “challenge" (Table 1), with further indicates an absence of priming whatever the combination of strains used.

**Table 1:** Parametric survival analysis of the effect of fungal priming in ants.

	Early fungal challenge			Late fungal challenge		
*Effect*	d.f.	χ^2^	*P*-value	d.f.	χ^2^	*P*-value
Priming	2.1	2.8	0.3	2.1	0.9	0.7
Challenge	1.3	25.6	<0.0001	1.2	12.65	<0.001
Priming x Challenge	2.1	0.6	0.8	2	2.6	0.3

*Summary table*		*Z*	*P*-value		*Z*	*P*-value
Effect of priming: homologous combinations						
C-S2 vs S2-S2		0.3	0.8		1.3	0.2
C-S3 vs S3-S3		1.4	0.2		0.4	0.7
Effect of priming: heterologous combinations						
C-S2 vs S3-S2		0.9	0.4		0.3	0.7
C-S3 vs S2-S3		1.5	0.1		0.6	0.5
Homologous versus heterologous combinations						
S2-S2 vs S3-S2		0.6	0.5		1.6	0.1
S3-S3 vs S2-S3		0.1	0.9		0.2	0.8

The ants were initially exposed to control buffer, low dose of *B. bassiana* strain S2 or low dose of *B. bassiana* strain S3 (factor: “priming") and subsequently challenged with high dose of either *B. bassiana* strain S2 or strain S3 (factor: “challenge"). The summary table of the model gives information on the effect of each combination of initial exposure (C = control, S2 = strain 2 or S3 = strain 3) and subsequent fungal challenge (S2 = strain 2 or S3 = strain 3). For example, the comparison “C-S2 vs S2-S2" examines whether the ants that were initially exposed to control buffer or to a low dose of strain S2 differed significantly in their survival when challenged with a high dose of strain S2.

Both fungal strains (S2 and S3) caused a highly significant mortality to the ants when applied at high doses in the challenge (Fig. 1 and Table 1; C-C vs C-S2, early challenge: d.f. = 3.9, *P*<0.0001, late challenge: d.f. = 3.3, *P*<0.001; C-C vs C-S3, early challenge: d.f. = 5.1, *P*<0.00001, late challenge: d.f. = 4.1, *P*<0.0001). The strains differed significantly in their virulence (Fig. 1 and Table 1; effect of strain used for the challenge, early challenge: χ^2^ = 25.6, d.f. = 1.3, *P*<0.0001, late challenge: χ^2^ = 12.65, d.f. = 1.2, *P*<0.001), with strain S3 inducing a higher mortality (Fig. 1). Overall, 83% of the corpses of ants that had been subjected to the fungal challenge produced hyaline spores that are diagnostic of an infection with *B. bassiana*. In contrast, none of the corpses of ants that had been exposed to the control buffer produced hyaline spores.

## Discussion

Immune priming has been found in multiple groups of invertebrates, but the generality and adaptive significance of the phenomenon remain controversial [6]. Here, we found no evidence for immune priming in ants exposed to naturally occurring fungal pathogens. The initial exposure of *F. selysi* workers to a sublethal dose of *B. bassiana* had no significant effect on their individual resistance in a later challenge with a lethal dose of the same fungus. We detected no sign of priming when testing short or long time intervals between initial exposure and challenge, nor when using two strains of *B. bassiana* in homologous or heterologous combinations.

It is somewhat difficult to determine the reason for this absence of priming in ants exposed to fungal pathogen. First, priming may vary across pathogen types, as well as across host taxa. Several studies suggest that priming depends on the type of parasite, for example on the species of bacteria [10], and may be absent in some host species [13]. Second, priming may depend on experimental conditions, which include a large number of parameters such as the mode of exposure, the time between first exposure and challenge, the doses and virulence of the pathogen, the costs induced by the primary exposure, or the nutritional status and stress level of the hosts [36], [37]. For example, we might have failed to detect priming because the fungus did not succeed in crossing the cuticle during primary exposure and thus did not trigger the immune defences in the haemocoel, or because *B. bassiana* had relatively low virulence in *F. selysi* and caused only moderate mortality to the ants during the challenge (15 to 45% as compared to controls). On the other hand, we used naturally occurring pathogens and mode of exposure, in contrast to studies in which non-specific pathogens were injected into the insects. We can conclude that no immune priming occurred in the conditions that we tested, without generalizing to other conditions or pathogens. Overall, there is no reason to expect strong and ubiquitous priming across all conditions in all invertebrates, particularly when considering that early studies failed to detect evidence for specific memory [6].

Most previous evidence for individual priming and specific memory in insects involved the injection of killed or live bacteria in the host, followed by a challenge with a lethal dose of bacteria three to 22 days after the priming e.g. [7]–[10]. So far, evidence for individual priming in insects to fungi are limited to two cases. One involved *Drosophila melanogaster* injected with heat-killed spores from *B. bassiana* [9]. The other concerned small groups of termites primed by contact with an extremely diluted solution of spores from *M. anisopliae*, suggesting that the immune priming was elicited by a soluble substance in the solution rather than by the spores themselves [7]. These conditions are quite different to the ones tested here, which might explain the different outcomes.

An important characteristic of our study is that we used a natural host-pathogen system and that we exposed the ants to low but significant doses of live fungal spores [38]. It is thus conceivable that *B. bassiana* has developed means to evade the specific detection and immune response of its host during the natural process of infection, for example by interfering with the immune system [39]. Immune evasion has indeed been documented in many groups of pathogens and parasites [25], [39], including entomopathogenic fungi [40], [41]. This hypothesis remains speculative, however, particularly when considering the low rate of successful infection by the fungal pathogen following primary exposure.

An alternative hypothesis is that the ants did not show immune priming because they had efficient behavioural or chemical group-level defences preventing infection [32], [42]. Ants indeed use multiple collective defences to socially control fungal and bacterial infections [29], [31]. These defences comprise modulations of social interactions, allo-grooming, trophallactic exchanges and sharing of antibiotics e.g. [30], [35], [43], [44]. For example, allo-grooming might have permitted to remove many of the spores contacted during the period of primary exposure [44]. Moreover, there is evidence that collective defences are traded off against individual defences [32], [42], [45]. It is thus possible that the efficiency of collective defences against fungal pathogens has permitted ants to reduce their investment in individual immune priming.

One last fascinating possibility is that even if we did not detect priming at the individual level when all group members were exposed, it might still occur at the group level towards individuals that have not been themselves exposed. Indeed, naïve *Lasius neglectus* ants and *Zootermopsis angusticollis* termites showed higher resistance to the fungal pathogen *M. anisopliae* after having been in contact with nestmates previously exposed to a low dose of the pathogen applied dorsally [33], [34]. In ants, such social effects might be mediated by modulating the rate and chemical nature of trophallactic exchanges [35]. Hence, even if the primary exposure of ant workers to a pathogen does not improve their individual resistance in later encounters with the same pathogen, it might still increase the resistance of members of the social group that have not yet been exposed. The occurrence of such social immune priming of naïve nestmates deserves to be further investigated, as it might be an important component of disease resistance in social animals.

## Materials and Methods

We sampled *F. selysi* workers from a population located along the river Rhône between Sierre and Susten in central Valais, Switzerland. No specific permit was required to collect this ant species, which is not endangered or protected. *F. selysi* nests in the soil and forages for invertebrates above ground. In June 2009, we collected workers and brood from each of 21 single-queen colonies [46]–[48]. We kept the ants in plastic boxes (13.5 cm long×15 cm wide×5 cm high) lined with fluon GP1 (Whitford Plastics, Diez, Germany) to prevent ants from escaping. The ants were brought to the laboratory and maintained at 25°C under a 12 hours day/night cycle. Throughout the experiments, including the initial fungal exposure and subsequent fungal challenge, the workers had *ad libitum* access to water and a protein-rich jelly food made of honey, chicken egg and agar [49].

We tested priming in young workers that had been collected as pupae in field colonies and had hatched in the laboratory. These workers were two to three months old at the start of the experiment. We used the generalist fungal entomopathogen *B. bassiana*, which is a common natural pathogen of *F. selysi* in our study site [38]. *B. bassiana* produces asexual spores ( = conidia) that attach to the cuticle of the ants, where they germinate and form an appressorium [50]. The hyphae penetrates through the cuticle within three days, develops in the haemocoel, kills the insect within eight days and produces large numbers of external conidiophores [50], [51].

To test the effect of homologous and heterologous combinations of strains, we selected two strains of *B. bassiana* that were genetically distinct at the ITS1-5.8S-ITS2 nuclear ribosomal cistron, the mitochondrial EH1 gene and six microsatellite markers [strains S2 and S3; [38]. The two strains had been isolated from our study site in September 2006. Both strains are rare in the study population and caused significant mortality to the ants [38]. Previous exposure of larvae to the pathogen is possible but unlikely, as in an extensive survey we detected the presence of *B. bassiana* in only 17% of the field colonies [38]. We used the strains S1 and S2 in homologous and heterologous combinations, that is, we primed and challenged the ants with the same genetic strain and with alternative genetic strains, respectively. For each strain, we used conidia originating from one infected individual. We cultured the spores on a nutritive medium (Malt Extract Agar) for five days at 25°C and harvested them into sterile 0.05% Tween 20.

We first exposed groups of workers to sublethal doses of fungal spores over a six-day period. Social interactions such as allo-grooming were possible during this period. Hence, this initial exposure in groups could trigger individual as well as social immune priming, if any. For each of the 21 colonies, we formed three groups of 40 young workers. We placed these workers in three large Petri dishes (9 cm diameter) lined with fluon and containing a filter paper on which the ants could walk freely. One of the three groups was exposed to *B. bassiana* strain S2, the other to *B. bassiana* strain S3 and the last one to control buffer. We adjusted the fungal dose (1.2×10^6^ spores in total) to be just below the one (4×10^6^ spores) causing a 50% mortality when applying a fungal pathogen of similar virulence on a filter paper [30], [38], [48]. As in other priming experiments, we wanted to expose the ants to the pathogen without causing significant illness or mortality. On the first day of the exposure period, we applied 500 µL of *B. bassiana* spore solution at low concentration (8×10^5^ conidia/ml) or 500 µL of 0.05% Tween 20 control buffer on the filter paper. We repeated these applications two days and four days after the beginning of the exposure period. On the seventh day, we removed the filter papers from the Petri dishes. Hence, ants were exposed to the fungus by walking on a filter paper harbouring sublethal doses of fungal spores for a period of six days.

We challenged individual workers with a lethal dose of fungal pathogen either eight or 16 days after the beginning of the six-day period of primary exposure (“early" and “late" fungal challenges, respectively). We applied 2 µL of spore solution (2.6×10^8^ conidia/ml) on the thorax of each individual ant. For each of the 21 colonies and each of the two time intervals between exposure and challenge, we challenged five randomly sampled individuals of each initial treatment (primary exposure to control, *B. bassiana* S2 and *B. bassiana* S3) with the strain S2, and five other individuals with the strain S3. To assess the baseline mortality of workers in absence of pathogens, five additional individuals that had been primarily exposed to control buffer received 2 µL of control buffer in the challenge phase.

After the secondary challenge, the ants were kept in isolation in small Petri dishes (3.5 cm diameter) containing a moist filter paper. We monitored the survival of the workers daily over eight days. To assess if the mortality was due to an infection by *B. bassiana*, we surface-sterilized all corpses by dipping them in 70% alcohol for a few seconds to facilitate wetting of the cuticle, placing them in 1% sodium hypochlorite for one minute and rinsing them three time in sterile water, as described in [52]. We then placed the corpses in tubes with wet cotton wool at 25°C and monitored the emergence of diagnostic hyaline spores for 30 days [52].

To assess the effect of the initial exposure to the low dose of fungal spores, we monitored the survival of all individuals not subjected to the “early" secondary challenge until day 16 (see above; *n* = 1260 exposed and 525 control ants, respectively). Thirty-five of these 1785 individuals (2%) died during this period. The mortality was not significantly different between exposed and control groups (Kruskal-Wallis test: χ^2^ = 1.2, d.f. = 2, *P* = 0.6). We surface-sterilized all corpses and checked them for spore production as described above. One of the corpses from the exposed group produced spores typical to an infection by *B. bassiana*.

### Statistical analyses

We compared the survival of primed and control workers in separate analyses for the early and late fungal challenges, respectively. The survival analysis was based on parametric regression models, using a Weibull distribution, as implemented in the *survreg* function of the software R [53]. We built a model with two fixed factors: “priming" (primary exposure to either control buffer, low dose of *B. bassiana* strain S2 or low dose of *B. bassiana* strain S3) and “challenge" (secondary challenge with high doses of either *B. bassiana* strain S2 or *B. bassiana* strain S3). We included the colony of origin as a random factor. The effects of the factors “priming", “challenge" and their interaction were evaluated using a chi-square likelihood ratio test. We sequentially removed non-significant terms. The summary table of the model permitted us to further compare the effects of each combination used for initial exposure (Control buffer, strain S2 or strain S3) and later fungal challenge (strain S2 or strain S3). To evaluate the virulence of each fungal strain, we run another model on the survival of workers that had been first exposed to control buffer and later challenged with either control buffer, *B. bassiana* strain S2 or *B. bassiana* strain S3.
